# The Impact of Metformin Use with Survival Outcomes in Urologic Cancers: A Systematic Review and Meta-Analysis

**DOI:** 10.1155/2021/5311828

**Published:** 2021-10-08

**Authors:** Xiangyang Yao, Haoran Liu, Hua Xu

**Affiliations:** ^1^Department of Urology, Tongji Hospital, Tongji Medical College, Huazhong University of Science and Technology, Wuhan, China; ^2^Department of Urology, The First Affiliated Hospital of Anhui Medical University, Institute of Urology, Anhui Medical University, Hefei, China

## Abstract

**Background:**

Conflicting results exist between the potential protective effects of metformin and the prognosis of urologic cancers. This meta-analysis summarized the effects of metformin exposure on the recurrence, progression, cancer-specific survival (CSS), and overall survival (OS) of the three main urologic cancers (kidney cancer, bladder cancer, and prostate cancer).

**Methods:**

We systematically searched PubMed, Embase, Web of Science, Wanfang, and China National Knowledge Infrastructure databases (January 2010 to December 2019), which identified studies regarding metformin users and nonusers with urologic cancers and extracted patient data. A random effect model or fixed effect model was used to analyze hazard ratios (HRs) and 95% confidence intervals (CIs).

**Results:**

Among the 1883 confirmed studies, 27 eligible studies were identified, including 123,212 participants. In prostate cancer, patients using metformin have significant benefits for recurrence (HR = 0.74; 95% CI: 0.61-0.90; *P* = 0.007; *I*^2^ = 56%), CSS (HR = 0.74; 95% CI: 0.61-0.91; *P* = 0.002; *I*^2^ = 79%), and OS (HR = 0.76; 95% CI: 0.65-0.90; *P* < 0.001; *I*^2^ = 86%). Moreover, further subgroup analysis showed that the beneficial effects of metformin may be more significant for patients receiving radical radiotherapy. For kidney cancer, metformin was beneficial for progression (HR = 0.80; 95% CI: 0.65-0.98; *P* = 0.14; *I*^2^ = 46%). Analysis revealed that the effect of metformin on the overall survival of kidney cancer patients may be related to nationality (American: HR = 0.76; 95% CI: 0.59-0.98; *P* = 0.88; *I*^2^ = 0%). For bladder cancer, no obvious benefits of metformin use were identified. However, subgroup analysis indicated that metformin may improve the recurrence of bladder cancer, but this improvement was only found in patients with a median follow-up time of more than 4 years (HR = 0.43; 95% CI: 0.28-0.67; *P* = 0.61; *I*^2^ = 0%).

## 1. Introduction

Due to the aging of the population and changes in people's lifestyles, the prevalence of diabetes is increasing substantially. It is estimated that diabetes will affect approximately 366 million people worldwide by 2030 [1]. Moreover, the microvascular and neurological complications impose a considerable economic burden on society. In recent years, as research on diabetes continues to expand, a number of studies have shown a strong association between diabetes and the risk of cancers [2]. Extensive epidemiological data have suggested a significant correlation between type 2 diabetes mellitus (DM2) and a variety of cancers [3]. Several cohort studies demonstrated that the incidence and mortality of cancers in diabetic patients are increasing [4–7]. In particular, prostate, lung, colorectal, pancreatic, and breast cancer have an association with DM2. Increased risks of kidney and urothelial carcinogenesis have also been reported [8, 9]. However, metformin, the most widely used first-line antidiabetic drug for type II diabetes, has been proposed to reduce the incidence of cancer and cancer-related mortality in patients with DM2 [10]. Both in vivo and in vitro studies have indicated that metformin has anticancer activity. The effect of reducing hepatic glucose output [11], reducing insulin resistance [12], and decreasing inflammatory responses [13] may inhibit the growth of cancer cells and ultimately lead to a reduction in the risks of solid tumors. AMPK signaling is considered to be the main mechanism by which metformin exerts an anticancer effect [14]. Furthermore, the AMPK signaling pathway is related to metabolic diseases, including hypertension, diabetes, and obesity [15], which may be the theoretical basis for DM2 patients taking metformin to reduce the risk of cancer.

The anticancer benefits of metformin in a number of cancers, including lung cancer, breast cancer, pancreatic cancer, and colorectal cancer, have been demonstrated in several meta-analyses [16–18]. Nevertheless, only a limited number of previous meta-analyses evaluated the survival effect (including overall survival and cancer-specific survival) of metformin for urologic malignancies [19], and controversy exist among previous studies on individual tumor types. In regard to prostate cancer, data from cohort studies of metformin use for radical prostatectomy revealed a nonsignificant reduction in the risk of biochemical recurrence [20] and an increase in the prostate cancer-specific survival [21]. Furthermore, Ahn et al. demonstrated that metformin may have no impact on recurrence or progression in patients with bladder cancer and diabetes [22]. The results regarding recurrence reported by Hakimi et al. and cancer-specific survival reported by Nayan et al. were also inconsistent with previous results for kidney cancer [23, 24].

Our goal was to conduct a systematic review to clarify the effect of metformin use on recurrence, progression, cancer-specific survival (CSS), and overall survival (OS) in the field of urologic oncology, particularly in patients with prostate cancer, bladder cancer, or kidney cancer.

## 2. Materials and Methods

### 2.1. Search Strategy and Study Selection

The PubMed, Embase, Web of Science, Wanfang, and China National Knowledge Infrastructure databases were searched to identify relevant studies investigating the relationship between the use of metformin and recurrence/progression/survival outcomes of patients with urologic cancers. The search strategy consisted of various combinations of the following terms: [“metformin” OR “biguanides”] and [“prostate cancer”/“kidney cancer”/“bladder cancer” OR “prostate carcinoma”/“kidney carcinoma”/“urothelial carcinoma”]. We also identified bibliographies of selected original studies and review articles. The protocol was registered in the International Prospective Register of Systematic Reviews database (PROSPERO: CRD42020193201).

### 2.2. Inclusion and Exclusion Criteria

Eligible studies fulfilled the following criteria: (1) randomized, controlled trials and nonrandomized studies that considered the association between metformin use and no metformin use in patients with urologic cancers and diabetes and (2) studies that reported data on at least one outcome of recurrence, progression, cancer-specific survival, and overall survival for individual types of cancer. In addition, exclusive criteria were as follows: (1) study not related to urologic cancers, (2) duplicate or invalid data, (3) incomplete data or unclear outcome effect, and (4) unable to provide hazard ratios and 95% confidence interval.

### 2.3. Data Collection and Quality Assessment

All retrieved studies underwent a qualification assessment, and the full text was obtained when information in the title or abstract was insufficient. If there were multiple publications for the same research or the patients studied were included in overlapping studies, we selected the publication that had the most complete information or the latest publication date. For each identified study, two independent researchers extracted and cross-checked the study information. When disagreements arise, we try to achieve maximum consensus by negotiating with another author. Information on the first author, publication year, patient sex, total subjects included, study location, study population, metformin exposure definition, median follow-up time, comparison groups, adjusted variables, clinical stage, treatments administered, and HRs and 95% CIs for recurrence, progression, CSS, and OS in both univariate and multivariate analyses were extracted into a predesigned table. The Newcastle–Ottawa Scale (NOS) was used to assess the methodological quality of all eligible studies.

### 2.4. Statistical Analysis

HRs or relevant data for each cancer type were extracted from the tables and figures of eligible studies. If enough data for each type of cancer was obtained, then a meta-analysis was conducted. The significance of the pooled HR was determined by the *Z* test, and *P* < 0.05 was considered statistically significant. Cochran's *χ*^2^-based *Q* test and *I*^2^ statistics were used to assess the heterogeneity among studies [25]. If *P* > 0.10 or *I*^2^ < 50%, there was no heterogeneity, and the fixed effect model was used to calculate the pooled HRs [26]; otherwise, the random effect model was performed [27]. For the definition of metformin exposure, patients who did not receive metformin treatment were defined as the “no-metformin” group (including some patients who received other medications), whereas those who received metformin were defined as the “metformin” group. Publication bias was evaluated by visually observing the asymmetry of Begg's funnel plot. RevMan software version 5.4 (Cochrane, London, UK) was used to create a risk-of-bias graph and summary.

### 2.5. Risk of Bias Assessment

**Figure 1:**
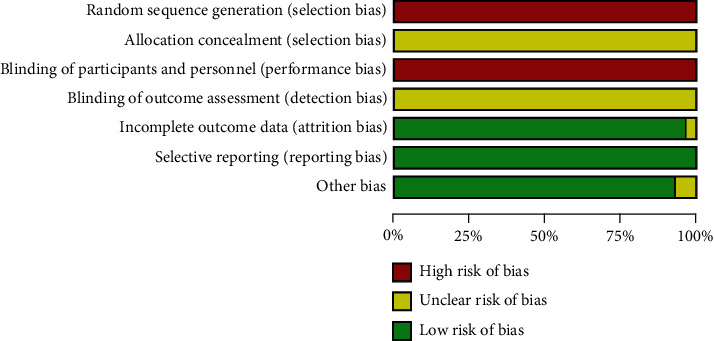
Risk of bias in the included studies.

**Figure 2:**
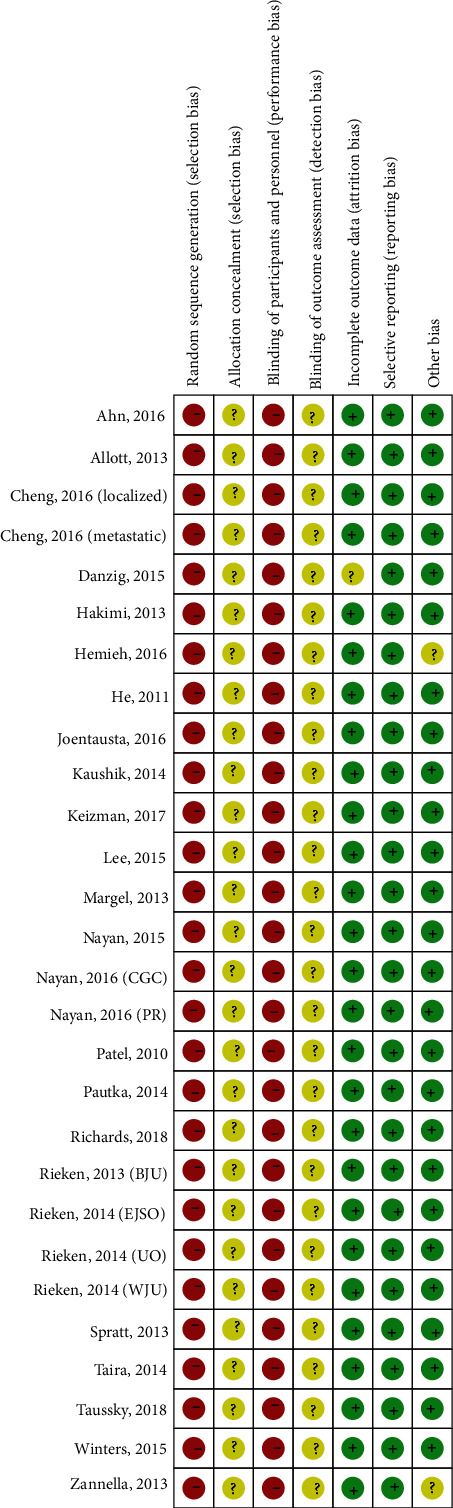
Risk of bias summary.

All articles do not use random sequence generation methods but are based on the generation methods of intervention measures, which have a high risk of bias. No studies reported on allocation concealment. Since all included studies did not use a placebo, all studies were assessed as having a high risk of bias in blinding of the participants and personnel. No studies reported on the blinding of outcome assessment. One study with missing persons or unreported reasons was assessed to have a high risk of incomplete outcome data. Except for two studies, the remaining studies were assessed to have a low risk of bias in other sources of bias domains, as they reported statistical homogeneity in the characteristics of participants between the groups at baseline (Figures 1 and 2).

## 3. Results

### 3.1. Characteristics of Included Studies

**Figure 3:**
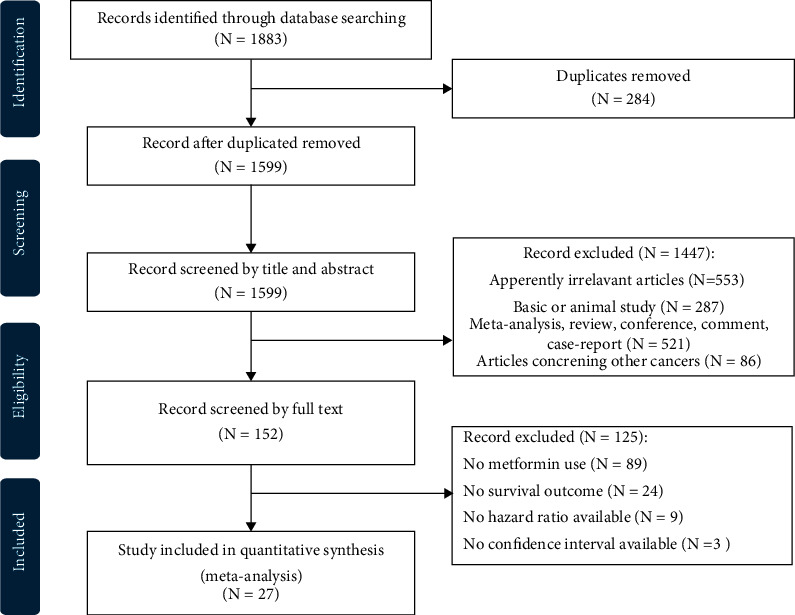
PRISMA study selection diagram.

We identified a total of 1883 records and excluded 1856 after browsing the title, abstract, or full text. Finally, 27 eligible articles were identified, including 123,212 participants [20–24, 28–49]. All selected studies were retrospective cohort studies. The PRISMA study selection diagram is shown in Figure 3. The characteristics of the included cohort studies are listed in Table 1. Most of the selected studies evaluated the recurrence, progression, CCS, and OS of metformin in one of three cancer types: kidney, bladder, and prostate cancer.

### 3.2. Recurrence

**Figure 4:**
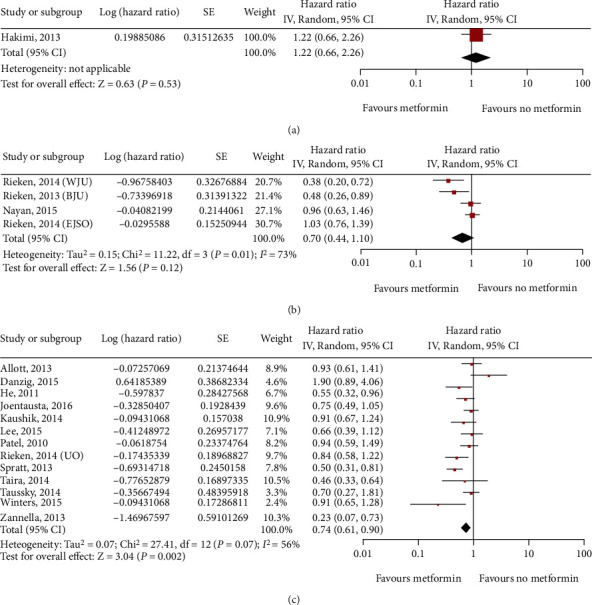
Forest plot of the recurrence of metformin use in patients with three main types of urinary system cancer: kidney cancer (a); bladder cancer (b); prostate cancer (c).

**Figure 5:**
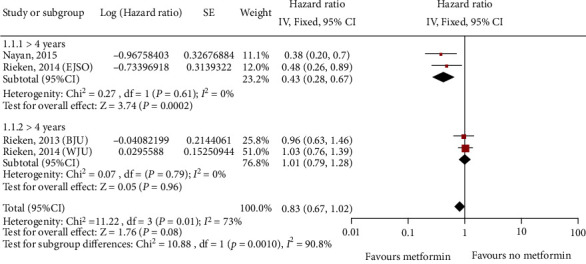
Forest plot of bladder cancer recurrence in different treatment groups based on metformin use.

One study on kidney cancer (784 patients), five studies on bladder cancer (7356 patients), and seven studies on prostate cancer (8127 patients) assessed recurrence. A multivariate analysis indicated that metformin did not significantly improve the recurrence rate of kidney cancer (HR = 1.22; 95% CI: 0.66-2.26; *P* = 0.53; Figure 4(a)) or bladder cancer (HR = 0.70; 95% CI: 0.44-1.10; *P* = 0.01; *I*^2^ = 73%; Figure 4(b)), but it significantly improved in prostate cancer (HR = 0.74; 95% CI: 0.61-0.90; *P* = 0.007; *I*^2^ = 56%; Figure 4(c)). As kidney cancer was evaluated in only one study involving 784 patients, a meta-analysis of this result was not possible. Moreover, metformin does not seem to have an influence on recurrence in this study. In addition, by analyzing the data in the table, we found that metformin was associated with differences in the median follow-up time for bladder cancer in the five studies. Therefore, we found that metformin had a benefit on the recurrence of bladder cancer, although the finding was limited to studies with a median follow-up time of more than 4 years (HR = 0.43; 95% CI: 0.28-0.67; *P* = 0.61; *I*^2^ = 0%; Figure 5).

### 3.3. Progression

**Figure 6:**
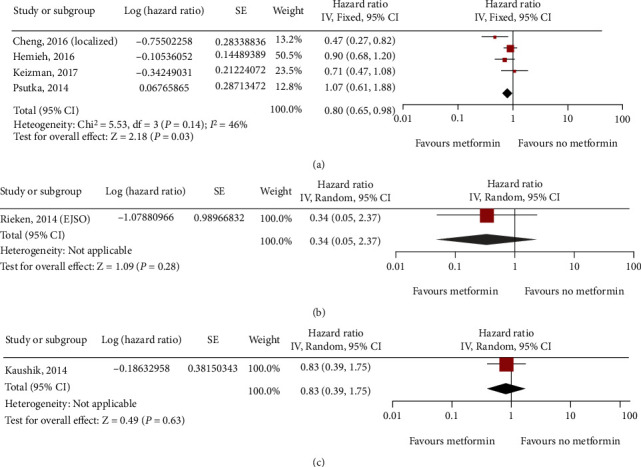
Forest plot of the progression of metformin use in patients with three main types of urinary system cancer: kidney cancer (a); bladder cancer (b); prostate cancer (c).

Five studies on kidney cancer (7356 patients), two studies on bladder cancer (1680 patients), and one study on prostate cancer (885 patients) evaluated progression. In the corresponding multivariate analysis, a benefit of metformin use was observed in kidney cancer (HR = 0.80; 95% CI: 0.65-0.98; *P* = 0.14; *I*^2^ = 46%; Figure 6(a)) when the fixed effect model was applied. Bladder cancer (HR = 0.34; 95% CI: 0.05-2.37) and prostate cancer (HR = 0.83; 95% CI: 0.39-1.75; Figures 6(b) and 6(c)) were only evaluated in one study each, and there was a certain degree of bias. Therefore, a meta-analysis could not be performed for this outcome.

### 3.4. Cancer-Specific Survival

**Figure 7:**
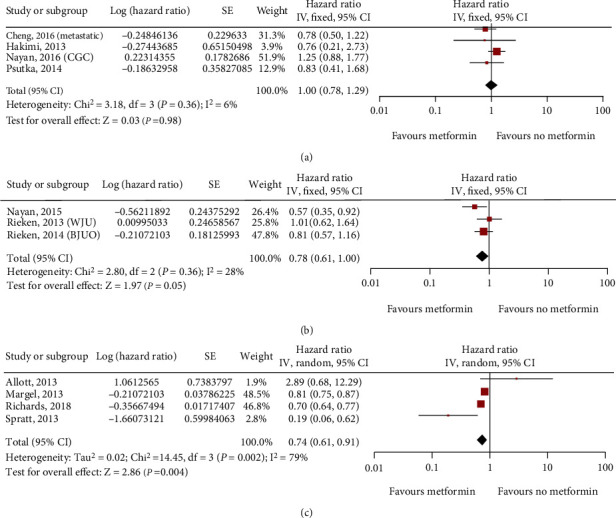
Forest plot of the cancer-specific survival (CSS) of metformin use in patients with three main types of urinary system cancer: kidney cancer (a); bladder cancer (b); prostate cancer (c).

Five studies on kidney cancer (3283 patients), four studies on bladder cancer (5168 patients), and four studies on prostate cancer (91869 patients) assessed cancer-specific survival. After multivariate analysis, we suggested that the use of metformin did not significantly improve the CSS of kidney cancer (HR = 1.00; 95% CI: 0.78-1.29; *P* = 0.36; *I*^2^ = 6%; Figure 7(a)), but it significantly improved in prostate cancer (HR = 0.74; 95% CI: 0.61-0.91; *P* = 0.002; *I*^2^ = 79%; Figure 7(c)). Moreover, bladder cancer showed a borderline improvement in CSS (HR = 0.78; 95% CI: 0.61-1.00; *P* = 0.25; *I*^2^ = 28%; Figure 7(b)).

### 3.5. Overall Survival

**Figure 8:**
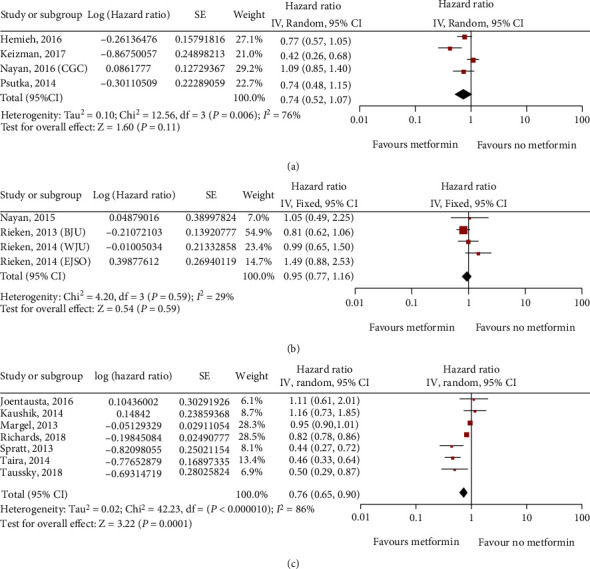
Forest plot of the overall survival (OS) of metformin use in patients with three main types of urinary system cancer: kidney cancer (a); bladder cancer (b); prostate cancer (c).

Six studies on kidney cancer (8127 patients), four studies on bladder cancer (5168 patients), and seven studies on prostate cancer (98438 patients) assessed overall survival (OS). The multivariate analysis demonstrated that the use of metformin seemed to be associated with significant improvements in OS for prostate cancer (HR = 0.76; 95% CI: 0.65-0.90; *P* < 0.001; *I*^2^ = 86%; Figure 8(c)). However, a relationship was not found between metformin and the OS for kidney cancer (HR = 0.74; 95% CI: 0.52-1.07; *P* = 0.06; *I*^2^ = 76%; Figure 8(a)) or bladder cancer (HR = 0.95; 95% CI: 0.77-1.16; *P* = 0.24; *I*^2^ = 29%; Figure 8(b)).

**Figure 9:**
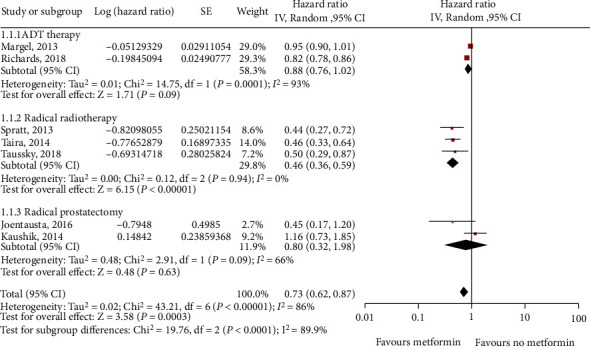
Forest plot of overall survival (OS) for prostate cancer (different treatment) with metformin use.

**Figure 10:**
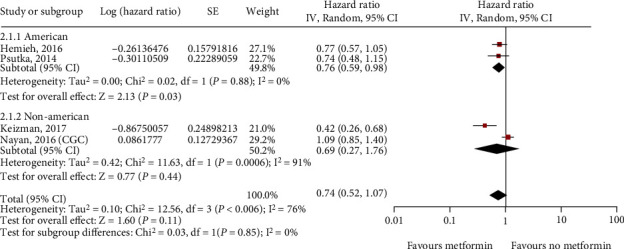
Association between metformin intake and overall survival (OS) subgrouped by ethnicity in kidney cancer.

Subsequently, we analyzed the effects of metformin on prostate cancer patients receiving different treatments and found a significant association between the primary type of treatment and the efficacy of metformin on OS. For patients receiving radical radiotherapy, metformin had significant benefits on OS, whereas metformin had no obvious benefits for patients undergoing radical prostatectomy and androgen deprivation therapy (ADT) (Figure 9). Moreover, an analysis of the sensitivity of patients with kidney cancer of different nationalities to the efficacy of metformin indicated that the overall survival of Americans was significantly improved after taking metformin, whereas there was no improvement in overall survival for non-Americans (Figure 10).

## 4. Discussion

Our systematic review showed that in urologic cancers, metformin use might be associated with a significant improvement in the recurrence, CSS and OS of prostate cancer, and the progression of kidney cancer. But no clear evidence has been found that it was associated with the progression of prostate cancer or the recurrence, CSS, or OS of kidney cancer and bladder cancer. Therefore, metformin was likely to be an effective adjuvant, especially in prostate cancer.

Metformin has recently attracted increasing attention and research interest due to its potential antitumor effects. However, the results of these studies have been inconsistent. After analyzing the OS and CSS of kidney cancer patients with diabetes, Li et al. showed that the use of metformin was beneficial to the prognosis of kidney cancer patients [50]. Nevertheless, Nayan et al. analyzed the OS, PFS, and CSS of kidney cancer patients with diabetes and found that there was no obvious association between metformin use and any survival outcome [51]. Similarly, studies by Nayan et al. [36] and Rieken et al. [34] demonstrated that the use of metformin could prolong the recurrence of bladder cancer. On the contrary, Ahn et al. [22] did not report this protective effect. Therefore, our study is the first comprehensive meta-analysis and systematic review of existing research that focuses on the use of metformin for the prevention and treatment of urologic cancers. Through an analysis of 27 studies, we found that metformin was significantly beneficial in prostate cancer. Our analysis suggested that in prostate cancer, the beneficial effects of metformin may be more significant for patients receiving radical radiotherapy. The reason for this phenomenon may be related to the role of the AMPK pathway in regulating the response of cells to radiation therapy [52]. In bladder cancer, we believed that the beneficial effect of metformin was limited to patients with a median follow-up time of more than 4 years, indicating that metformin as a cancer adjuvant may require a longer observation time and more comprehensive research. Furthermore, a subgroup analysis indicated that metformin had a protective effect on the incidence of bladder cancer in Americans. However, no such protection was observed for non-Americans, indicating that the protective effect of metformin on kidney cancer may be related to nationality.

The main advantage of our research was that it analyzed the latest and most comprehensive studies. In terms of collecting articles, we analyzed and sorted out the most important research on urologic cancers in the past decade, and a substantial number of patients (mainly kidney cancer, bladder cancer, and prostate cancer) were included. As for outcome analysis, we considered several outcomes (including recurrence, progression, CSS, and OS) and performed subgroup analysis. During data analysis, we strictly classified and sorted out univariate and multivariate analyses and mainly performed multivariate analysis. Despite these advantages, limitations in the research must be noted. First, differences in sample size, proportion of metformin patients, nationality, and follow-up time may result in heterogeneity among the various studies. We controlled for heterogeneity according to *I*^2^ through a random effect model. Second, most of the studies included in this meta-analysis only contained results for two to three outcomes. Due to the insufficient data of the outcomes, it was difficult for us to determine potential relationships for certain factors. In addition, many of the studies conducted either univariate or multivariate analyses, which could lead to insufficient data and bias when we strictly evaluated multivariate analysis. Third, we compared population heterogeneity. The control group was defined as “patients not using metformin” in some studies, but the control group may have included patients receiving any other hypoglycemic drugs. Therefore, these differences may lead to deviations in the therapeutic effect of metformin.

## 5. Conclusion

Despite these limitations, this study was still of great significance for the treatment and prognosis of patients with urologic cancers. In general, systematic reviews and meta-analyses have indicated that metformin has certain benefits for urologic cancers (prostate cancer), especially for patients receiving cancer radiotherapy. Of course, for patients with kidney cancer, we found that the prognosis may be related to nationality. Additionally, research on the recurrence of bladder cancer helped further elucidate that there may be a certain correlation between the prognosis of cancer and the dose and duration of metformin, which may require further research to verify. Therefore, a large amount of research is needed to confirm the prognostic benefits and evaluate the possibility of metformin as an adjuvant in the wider cancer population.

## Figures and Tables

**Table 1:** Characteristics of included studies.

Tumor group	Author (year) [ref.]	Treatment	Stage/other restrictions	Sample size (met/total)	Study location	Setting	DM	Non-DM	Recurrence	Progression	CSS	OS	Definition of metformin exposure	Median follow-up (months)	Adjusting variables	NOS score
Renal cell carcinoma	Hakimi et al. (2013) [23]	Partial/radical nephrectomy	T2–T3 N0 M0	55/784	USA	H	✓	✓	✓	✓	✓	✓	At surgery	41	Age, BMI, race, etc.	6
	Nayan et al. (2016) [24] (PR)	Not specified	Localized	NA/613	Canada	H	X	✓	X	X	✓	✓	At diagnosis	40	Age, sex BMI, nuclear grade	8
	Psutka et al. (2015) [28]	Partial/radical nephrectomy	Localized	83/200	USA	H	✓	X	X	✓	✓	✓	90 days before surgery	97	Mayo clinic, clinic stage, size, etc.	8
	Cheng et al. (2016) [29] (localized)	Partial/radical nephrectomy	T1–T3 N1 M1	390/1528	Singapore	H	X	✓	X	✓	✓	✓	At diagnosis	43	Not given	6
	Cheng et al. (2016) [29] (metastatic)	Partial/radical nephrectomy	T1–T3 N1 M1	390/1528	Singapore	H	X	✓	X	✓	✓	✓	At diagnosis	43	Not given	6
	Keizman et al. (2016) [30]	Systemic therapy	Metastatic	52/108	Israel	H	✓	X	X	✓	X	✓	At diagnosis	NA	Age, sex, race, ECOG status, histology, etc.	6
	Hamieh et al. (2017) [31]	Partial/radical nephrectomy	Metastatic	218/4736	USA	H	✓	✓	X	✓	X	✓	At diagnosis	NA	Age, gender race, previous therapy, etc.	8
	Nayan et al. (2017) [32] (CGC)	Partial/radical nephrectomy	T1–T3 N1 M0	82/158	Canada	H	X	✓	X	X	✓	✓	At surgery	43	Not given	8
Urothelial carcinoma	Ahn et al. (2016) [22]	TURBT	pTa–pT1	127/645	Korea	H	✓	✓	✓	✓	X	X	At diagnosis	46	Age, sex, BMI, DM, hypertension, tumor size, smoking, etc.	7
	Rieken et al. (2013) [33] (BJU)	TURBT	pTa–pT1 N0 M0	43/1035	USA and Europe	H	X	✓	✓	✓	✓	✓	At surgery	64	Age, tumor stage and grade, tumor size, etc.	8
	Rieken et al. (2014) [34] (UO)	Radical surgery	pT0-pT4 M0	80/1382	USA and Europe	H	X	✓	✓	X	✓	✓	At diagnosis	34	Age, sex, BMI, smoking, tumor stage and grade, etc.	8
	Rieken et al. (2014) [35] (EJSO)	Radical surgery	pT0-pT4 M0	194/2330	USA and Europe	H	X	✓	✓	X	✓	✓	At surgery	36	Age, sex, BMI, tumor stage, etc.	6
	Nayan et al. (2015) [36]	Radical surgery	pT0-pT4 N1 M0	39/421	Canada	H	X	✓	✓	X	✓	✓	At diagnosis	50	Age, sex, BMI, GFR, etc.	8
Prostate carcinoma	Danzig et al. (2015) [20]	Prostatectomy	Localized	98/767	USA	H	✓	X	✓	X	X	X	At surgery	27	Not given	6
	Allott et al. (2013) [21]	Prostatectomy	Localized	155/369	USA	H	✓	X	✓	X	✓	X	At surgery	59/73^b^	Age, sex, race, BMI, preoperative PSA, etc.	8
	Patel et al. (2010) [37]	Radical prostatectomy	Localized	112/616	USA	H	X	✓	✓	X	X	X	At diagnosis	NA	Age, clinical stage, preoperative PSA, etc.	7
	He et al. (2011) [38]	Prostatectomy/radical radiotherapy	Localized	NR/233	USA	H	X	✓	✓	X	X	X	At diagnosis	55	Age, sex, Gleason score, clinical stage	8
	Margel et al. (2013) [39]	Prostatectomy/ADT	Localized^a^/≥66 years old	1251/3837	Canada	P	✓	X	X	X	✓	✓	Cumulative exposure	56	Age, urban, Gleason score, etc.	8
	Spratt et al. (2013) [40]	Radical radiotherapy	Localized	157/319	USA	H	✓	X	✓	X	✓	✓	At diagnosis or after	104	Age, Gleason score, tumor stage, etc.	8
	Zannella et al. (2013) [41]	Radical radiotherapy	Localized	114/504	Canada	H	✓	✓	✓	X	X	X	At the time of radiotherapy	82	Age, PSA value, Gleason score, follow-up time, etc.	5
	Kaushik et al. (2014) [42]	Prostatectomy	Localized	323/885	USA	H	✓	X	✓	✓	X	✓	3 months before surgery	61	Age, BMI, Gleason score, stage, margin, etc.	7
	Rieken et al. (2014) [43] (WJU)	Prostatectomy	Localized^a^/≥66 years old	287/6486	USA and Europe	H	X	✓	✓	X	X	X	At surgery	25	Age, PSA value, Gleason score, lymph node metastasis, etc.	6
	Taira et al. (2014) [44]	Brachytherapy	Localized	126/2298	USA	H	✓	✓	✓	X	X	✓	Diagnosis to 3 months after	100	Age, follow-up years, PSA value, etc.	7
	Lee et al. (2015) [45]	Radical prostatectomy	T1–T4 N1 M0	209/746	Korea	H	X	✓	✓	X	X	X	3 months before surgery	43	Age, BMI, PSA, prostate volume, etc.	7
	Winters et al. (2015) [46]	Radical radiotherapy	Localized	366/1734	USA	H	✓	✓	✓	X	X	X	At diagnosis	41	Age, race, BMI, DM, etc.	7
	Joentausta et al. (2016) [47]	Radical prostatectomy	Localized	133/1314	Finland	H	✓	X	✓	X	X	✓	At diagnosis	103	Age, PSA level, Gleason score, tumor stage, etc.	8
	Richards et al. (2018) [48]	ADT	Localized	18940/87344	USA	H	✓	✓	✓	X	✓	✓	At diagnosis	24	Age, race, etc.	6
	Taussky et al. (2018) [49]	Radical radiotherapy	Localized	281/2441	Canada	H	✓	✓	✓	X	X	✓	At the time of radiotherapy	48	Age, CAPRA score, type of treatment	7

H: hospital; P: population; met: metformin; DM: diabetes mellitus; NOS: Newcastle–Ottawa Quality Assessment Scale for Cohort Studies; NA: not applicable; OS: overall survival; CSS: cancer-specific survival; BMI: body mass index; PSA: prostate-specific antigen; GFR: glomerular filtration rate; ECOG: electrocorticography; CAPRA: Cancer of the Prostate Risk Assessment; NA: not available. ^a^Data from subanalysis. ^b^Metformin/nonmetformin.

## Data Availability

The PubMed, Embase, Web of Science, Wanfang, and China National Knowledge Infrastructure databases were searched to identify relevant studies investigating the relationship between the use of metformin and recurrence/progression/survival outcomes of patients with urologic cancers.
